# Antioxidant and Anti-Inflammatory Activities of Pomegranate (*Punica granatum*) on *Eimeria papillata*-Induced Infection in Mice

**DOI:** 10.1155/2015/219670

**Published:** 2015-01-15

**Authors:** Omar S. O. Amer, Mohamed A. Dkhil, Wafaa M. Hikal, Saleh Al-Quraishy

**Affiliations:** ^1^Medical Laboratory Department, College of Applied Medical Sciences, Majmaah University, Al Majma'ah 11952, Saudi Arabia; ^2^Department of Zoology, Faculty of Science, Al-Azhar University (Assiut Branch), Assiut 71524, Egypt; ^3^Department of Zoology, College of Science, King Saud University, P.O. Box 2455, Riyadh 11451, Saudi Arabia; ^4^Department of Zoology and Entomology, Faculty of Science, Helwan University, Helwan 11795, Egypt; ^5^Parasitology Laboratory, Water Pollution Research Department, Environmental Division, National Research Center, Dokki, 12622 Giza, Egypt; ^6^Department of Biology, Faculty of Science, Tabuk University, P.O. Box 741, Tabuk 71491, Saudi Arabia

## Abstract

Coccidiosis is the most prevalent disease causing widespread economic loss, especially in poultry farms. Here, we investigated the effects of pomegranate peel extract (PPE) on the outcome of coccidiosis caused by *Eimeria papillata* in mice. The data showed that mice infected with *E. papillata* and treated with PPE revealed a significant decrease in the output of oocysts in their faeces by day 5 p.i. Infection also induced inflammation and injury of the jejunum. This was evidenced (i) as increases in reactive oxygen species, (ii), as increased neutrophils and decreased lymphocytes in blood (ii) as increased mRNA levels of inducible nitric oxide synthase (iNOS), Bcl-2 gene, and of the cytokines interferon gamma (IFN-*γ*), tumour necrosis factor-*α* (TNF-*α*), and interleukin-1*β* (IL-1*β*), and (iv) as downregulation of mucin gene MUC2 mRNA. All these infection-induced parameters were significantly altered during PPE treatment. In particular, PPE counteracted the *E. papillata*-induced loss of the total antioxidant capacity. Our data indicated that PPE treatment significantly attenuated inflammation and injury of the jejunum induced by *E. papillata* infections.

## 1. Introduction

Eimeriosis is a parasitic disease in which the parasites not only live inside the animal body but can also survive in wet moist conditions outside of the body. Animals that appear healthy shed the parasites in their stools. Unfortunately, in the right conditions, these parasites can quickly multiply within the intestinal tract causing tissue damage and inducing a severe local and systemic inflammatory response [1, 2], leading to diminished feed intake and nutrient absorption, reduced body-weight gain, dehydration, blood loss, and increased susceptibility to other diseases. Eimeriosis, therefore, causes huge economic loss in the fields of animal farming and milk and meat production [3].* Eimeria papillata* parasitizes mice and provides a convenient model for studying animal coccidiosis through its intracellular development within the mouse jejunum [4].

Medicinal plants have long been found to be useful in the development of new drugs and continue to play an important role in the drug discovery processes [5]. Such plants are relatively cheap and available and their uses are dependent on ancestral experience. The majority of people in developing countries remain dependent on traditional plants for healthcare [5].

Ancient Egyptians realized the benefits of pomegranate as a remedy for a variety of ailments [6] and, in recent times, pomegranate has been shown to have multiple beneficial effects, such as antiparasitic, hypolipidemic, hypoglycaemic, and antitumour activities [7]. Recently, Dkhil [8] reported the anthelmintic and the anticoccidial activity of pomegranate. The role of pomegranate in the regulation of gene expression due to eimeriosis has not, however, been studied before. The current study was designed to investigate the antioxidant activities of pomegranate, as well as its role in the expression of the inflammatory cytokines mRNA in the jejunum of mice infected with* Eimeria papillata*.

## 2. Materials and Methods

### 2.1. Preparation of the Pomegranate Peel Extract

*P. granatum* peels were obtained from fruits purchased from a local market in Riyadh, Saudi Arabia. The samples were authenticated by Dr. Jacob Thomas (Botany Department, College of Science, King Saud University, Saudi Arabia). Pomegranate peel extract was prepared according to the method described by Abdel Moneim [9], with some modification. Air-dried powder (100 g) of pomegranate peels was extracted by percolation at room temperature with 70% methanol and kept at 4°C for 24 h. The obtained extract was concentrated under reduced pressure (at a bath temperature of 50°C) and dried in a vacuum evaporator. The residue was dissolved in distilled water and used in this experiment.

### 2.2. Animals

Swiss Albino mice were inbred under specified pathogen-free conditions at facilities in the Zoology Department, King Saud University. Only male mice were used for the experiments. These were housed in plastic cages and received a standard diet and water ad libitum. The experiments were approved by the state authorities and followed Saudi Arabian law on animal protection.

### 2.3. Infection of Mice

Oocysts of* Eimeria papillata* were collected from the faeces of infected mice, surface-sterilized with sodium hypochlorite, and washed at least four times with sterile saline solution before oral inoculation as described previously by Dkhil et al. [10]. Each mouse was orally inoculated with 10^3^ sporulated oocysts of* E. papillata* suspended in 100 *μ*L sterile saline solution. Subsequently, fresh faecal pellets were collected every 24 h. The pellets collected from each mouse were weighed and the bedding was changed to eliminate reinfection. Oocyst output was measured as previously described by Dkhil et al. [10]. Faecal pellets were suspended in 2.5% (wt/vol) potassium dichromate and diluted in saturated sodium chloride for oocyst flotation. Oocysts were counted in a McMaster chamber and are expressed here as the number of oocysts per gram of wet faeces.

### 2.4. Experimental Design

The mice were divided into four groups with eight animals per group. The first group was gavaged with saline and served as the control, noninfected, group. The second group was inoculated by oral gavage with 100 *μ*L pomegranate peel extract (300 mg/kg) daily over 5 days. The dose and the route of injection were selected on the basis of the previous studies by Dkhil et al. [8, 11]. The third and fourth groups were infected with 100 sporulated oocysts of* E. papillata* and treated for five days with either sterile saline solution or pomegranate extract, respectively.

### 2.5. Neutrophils and Lymphocytes

At day 5 p.i. with* E. papillata*, blood was collected from all groups. Immediately following blood collection, 5 *μ*L of blood was used to prepare blood smears, which were stained with Giemsa stain for differential cell counts.

### 2.6. Reactive Oxygen Species and Total Antioxidant Capacity

For homogenization, part of the jejunum was weighed and homogenized immediately in order to prepare a 50% (w/v) homogenate in an ice-cold medium containing 50 mM Tris-HCl and 300 mM sucrose. The initial homogenate was centrifuged at 500 ×g for 10 min at 4°C. The supernatant (10%) was diluted with the Tris-sucrose buffer.

To determine the generation of reactive oxygen species (ROS), we used a modified assay for the intracellular conversion of nitro blue tetrazolium (NBT) to formazan by superoxide anion. Briefly, 200 *μ*L NBT (1.0 mg/mL) was added to the jejunal homogenate from each of the groups, followed by an additional period of incubation for 1 h at 37°C. Solutions were then treated with 100 *μ*L KOH (2 M). NBT reduced/g tissue expressed in nmol was determined through spectrophotometry. If radical-scavenging compounds were present in the tissue, less formazan blue would be formed, thus decreasing the absorption at 570 nm.

Total antioxidant capacity was assayed by the colorimetric technique using commercial kits (Biodiagnostic, Egypt). In brief, the method depends on the ability of antioxidants within the test sample to hydrolyse exogenously added hydrogen peroxide. After the reaction time the remaining H_2_O_2_ is determined colorimetrically by an enzymatic reaction which involves the conversion of 3,5,dichloro-2-hydroxybenzene sulfonate into a coloured product, the intensity of which is inversely proportional to the total antioxidant amount in the original sample [12].

### 2.7. Histology and Bcl-2 Immunohistochemistry

Small pieces of the jejuna were quickly removed and fixed in neutral buffered formalin. Following fixation, specimens were dehydrated, embedded in wax, and then sectioned to 5 *μ*m thicknesses. For histological examinations, sections were stained with haematoxylin and eosin. An immunolocalization technique for Bcl-2 was performed on 3-4 *μ*m thickness sections according to [13]. For negative controls, the primary antibody was omitted. In brief, mouse anti-Bcl-2 (diluted 1 : 200, Santa Cruz Biotechnology, Santa Cruz, CA, USA) was incubated with sections for 60 min. Primary antibodies were diluted in TBS (Tris buffered saline)/1% BSA (bovine serum albumin). Then a biotinylated secondary antibody directed against mouse immunoglobulin (Biotinylated Link Universal-DakoCytomation kit, supplied ready to use) was added and incubated for 15 min, followed by horseradish peroxidase conjugated with streptavidin (DakoCytomation kit, supplied ready to use) for a further 15 min incubation. At the sites of immunolocalization of the primary antibodies, a reddish to brown colour appeared after adding 3-amino-9-ethylcarbazole (AEC) (DakoCytomation kit, supplied ready to use) for 15 min. The specimens were counterstained with haematoxylin for 1 min and mounted using the Aquatex fluid (Merck KGaA, Germany). All sections were incubated under the same conditions with the same concentration of antibodies and at the same time; so the immunostaining was comparable between the different experimental groups.

### 2.8. Quantitative Real-Time PCR

Pieces of jejunum were aseptically removed, rapidly frozen, and stored in liquid nitrogen until use. Total RNA was isolated using Trizol (Invitrogen). RNA samples were treated with DNase (Applied Biosystems, Darmstadt, Germany) for at least 1 h and were then converted into cDNA by following the manufacturer's protocol using the reverse transcription kit (Qiagen, Hilden, Germany). Quantitative real-time PCR (qRT-PCR) was performed using the ABI Prism 7500HT sequence detection system (Applied Biosystems, Darmstadt, Germany) with SYBR green PCR master mix from Qiagen (Hilden, Germany). We investigated the genes encoding the mRNAs for the following proteins: interleukin-1*β* (IL-1*β*), tumour necrosis factor alpha (TNF-*α*), interferon-*γ* (IFN-*γ*), inducible nitric oxide synthase (iNOS), and mucin 2 (MUC2). All primer assays used for qRT-PCR were obtained commercially from Qiagen. PCRs were performed as previously described by Dkhil et al. [14].

### 2.9. Statistical Analysis

Results were expressed as the means ± standard deviation (SD). Data for multiple variable comparisons were analysed by one-way analysis of variance (ANOVA). For the comparison of significance between groups, Duncan's test was used as a post hoc test according to the statistical package program (SPSS version 17.0).

## 3. Results

The presence of infection was associated with clinical signs such as general weakness, poor performance, loss of appetite, and diarrhoea. These signs appear attenuated in the group pretreated with PPE. Infection of mice with* E. papillata* resulted in an output of about 310,000* E. papillata* oocysts per gram of faeces on day 5 p.i., as shown in Table 1. Treatment of mice with PPE extract significantly decreased the quantity of oocysts expelled, by about 55%.

**Figure 1:**
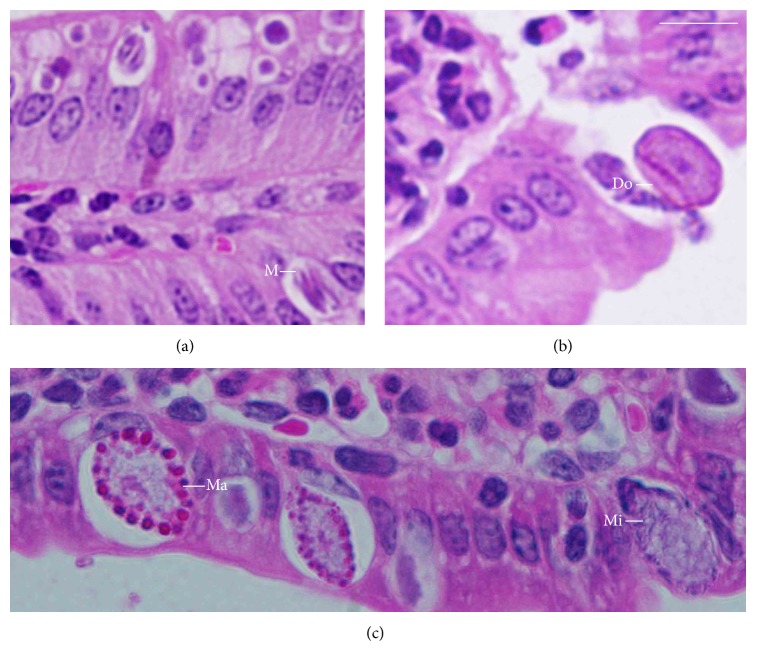
Different developmental stages of* E. papillata* in the jejunum of a mouse on day 5 p.i. (a, b, and c). Meront (M), microgamont (Mi), macrogamont (Ma), and developing oocyst (DO). See Table 1 for quantification. Bar: 25 *μ*m.

Next, we investigated whether the decreased expulsion of oocysts was due to the impaired development of* E. papillata* in the jejunum (Figure 1). In the infected group, we counted 95 ± 11 meronts, 64 ± 12 gamonts, and 31 ± 5 developing oocyst stages in the villi of the haematoxylin-eosin-stained sections (Table 1, Figure 1). These numbers were significantly decreased when mice were treated with PPE (Table 1). Remarkably, the total number of all parasitic stages decreased from 190 ± 14 to 110 ± 11 after PPE treatment (Table 1).

**Figure 2:**
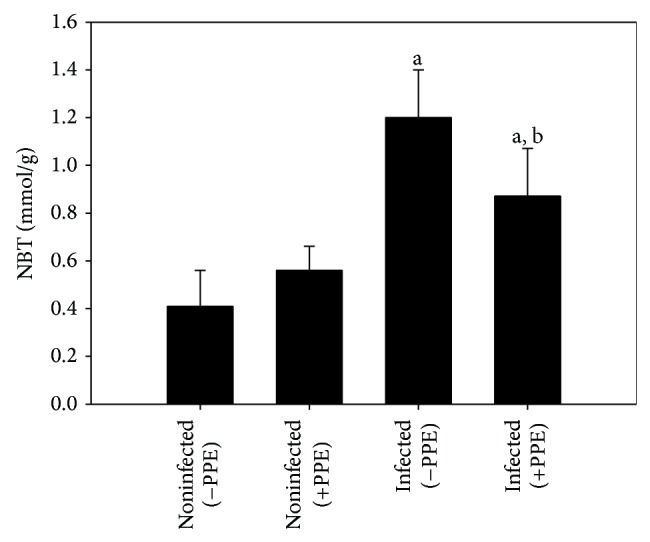
Reactive oxygen species (ROS) levels in jejunal homogenates of mice. Values are means ± SD. a: significant change at *P* < 0.01 with respect to noninfected (−PPE) mice. a, b: significant change at *P* < 0.01 with respect to infected (−PPE) mice.

**Figure 3:**
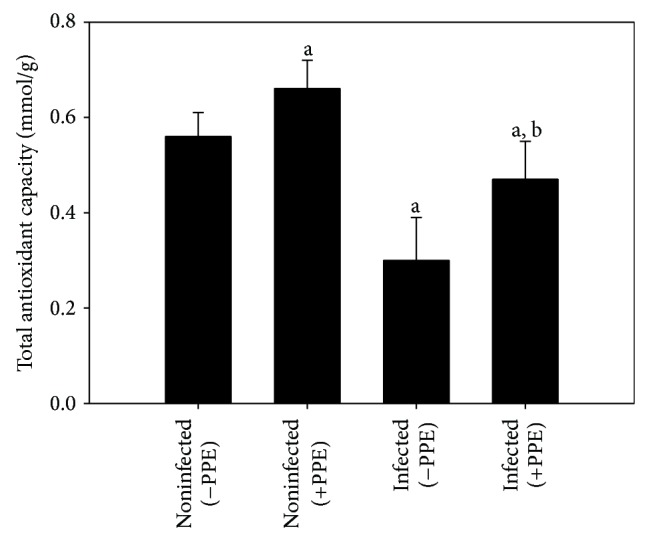
Total antioxidant capacity levels in jejunal homogenates of mice. Values are means ± SD. a: significant change at *P* < 0.01 with respect to noninfected (−PPE) mice. a, b: significant change at *P* < 0.01 with respect to infected (−PPE) mice.

*E. papillata* infection significantly (*P* < 0.05) induced in vivo production of oxygen free radicals in the jejunal tissues of mice as indicated by NBT assay (Figure 2). When mice were treated with pomegranate, the jejunal tissues were able to inhibit the reduction of NBT (Figure 2). Moreover, PPE was able to increase the reduced total antioxidant capacity due to* E. papillata* infection from 0.3 ± 0.09 to 0.47 ± 0.08 mmol/g (Figure 3).

**Figure 4:**
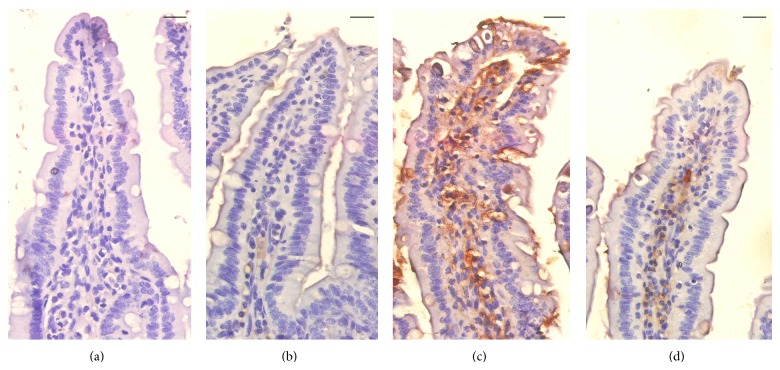
Immunohistochemical localization of Bcl-2 in the jejuna of mice. (a) Noninfected jejunum. (b) Noninfected PPE treated mouse jejunum. (c)* E. papillata* infected jejunum with increased number of Bcl-2 positive cells. (d) Infected treated mouse with decreased number of Bcl-2 positive cells. Bar: 25 *μ*m.

**Figure 5:**
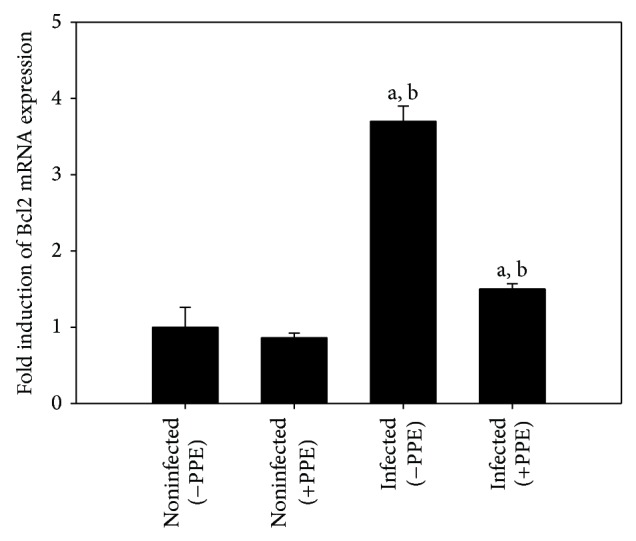
Quantitative RT-PCR analysis of Bcl-2 mRNA in the jejunum. Expression was analysed on day 5 p.i., normalized to 18S rRNA signals, with the relative expression given as fold increase compared to the uninfected control mice. Values are means ± SD. a: significant change at *P* < 0.01 with respect to noninfected (−PPE) mice. a, b: significant change at *P* < 0.01 with respect to infected (−PPE) mice.

Then, we checked the role of PPE in infection-induced apoptosis, through the histochemical staining of mice jejuna. Immunohistochemical investigation for Bcl-2 showed that PPE was able to decrease the immunoreactivity in the jejuna of mice infected with* E. papillata* (Figure 4). Also, PPE significantly improved the increased jejuna mRNA expression of Bcl-2 due to infection (Figure 5).

A significant systemic inflammatory response was initiated as a consequence of the infection, as revealed by the elevation in the number of neutrophils and a reduction in the number of lymphocytes (Table 2). Treatment with PPE, however, was associated with a significant amelioration in the relative percentages of neutrophils and lymphocytes (Table 2).

**Figure 6:**
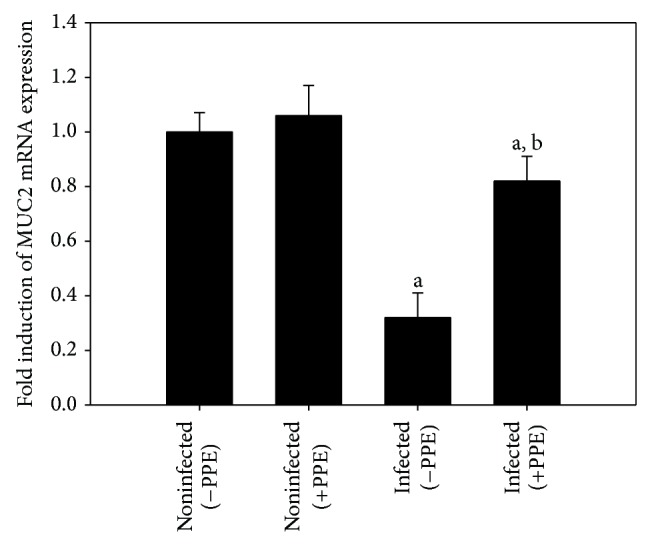
Quantitative RT-PCR analysis of MUC2 mRNA in the jejunum. Expression was analysed on day 5 p.i., normalized to 18S rRNA signals, with the relative expression given as fold increase compared to the uninfected control mice. Values are means ± SD. a: significant change at *P* < 0.01 with respect to noninfected (−PPE) mice. a, b: significant change at *P* < 0.01 with respect to infected (−PPE) mice.

In this study, the mRNA expression of MUC2 appeared significantly downregulated upon infection but significantly increased again after treatment with PPE (Figure 6).

**Figure 7:**
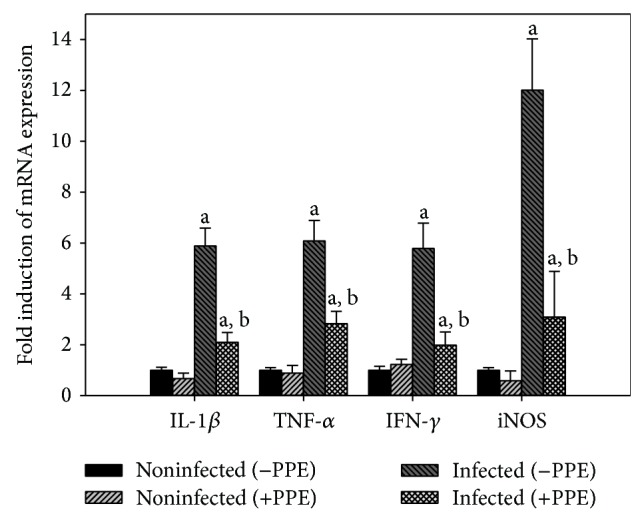
Quantitative RT-PCR analysis of TNF-*α*, iNOS, IFN-*γ*, and IL-1*β* in the jejunum. Expression was analysed on day 5 p.i., normalized to 18S rRNA signals, and relative expression is given as fold increase compared to the uninfected control mice. Values are means ± SD. a: significant change at *P* < 0.01 with respect to noninfected (−PPE) mice. a, b: significant change at *P* < 0.01 with respect to infected (−PPE) mice.

Also, the mRNA levels of iNOS and inflammatory cytokines, IL-1*β*, TNF-*α*, and IFN-*γ* (Figure 7), were upregulated after infection with* E. papillata*, whereas PPE significantly reduced the expression of these genes (Figure 7).

## 4. Discussion

Pomegranate is one of those candidate plants which have a chemoprotective effect and strong antioxidant potential [15]. The components of pomegranate contain compounds offering some impressive therapeutic applications [16]. Here, we show that pomegranate exhibits anticoccidial activity, evidenced as a significant lowering in the output of* E. papillata* oocysts within the faeces of the infected mice. This diminished output suggests that pomegranate impairs the development of parasites in the host before the relatively inert oocysts are formed and finally released. The fact that pomegranate possesses anticoccidial activity has also been reported by Dkhil [8], where it was able to significantly decrease* E. papillata* oocyst output in faeces of mice.

Elevated levels of ROS are an indication of the oxidative stress and cytotoxicity induced by the infection. Our results show, however, that ROS production was decreased significantly by pomegranate administration. A possible explanation is that, at the cellular level, the antioxidant effect of pomegranate may have an impact on the ROS levels induced by the infection by scavenging oxygen free radicals [17]. Increased production of ROS during infection leads to a rapid consumption and depletion of endogenous scavenging antioxidants. In this context, and in an effort to prevent or diminish oxidative stress induced damage, researchers are evaluating potential drugs to act as exogenous antioxidants, as well as scavengers to counter oxidative attack in these diseases.

Natural products have been used for dietary therapy for several millennia and some of these products allegedly exhibit significant antioxidant activity [18]. Also, the elevated levels of ROS during infection serve to facilitate pathogen clearance as well as contributing to signalling cascades related to inflammation, cell proliferation, and immune responses [19]. Moreover, ROS accumulation in host cells leads to cell apoptosis [20], whereas PPE could reduce this infection-induced increase in the number of apoptotic cells in the jejuna of mice infected with* E. papillata* [14]. Here, we showed also that PPE is able to lower the Bcl2 immunoreactivity in the infected jejunum.

In the present study, the percentage of neutrophils and lymphocytes was subject to significant alterations due to infection with the highly pathogenic* Eimeria*. This may relate to the critical role of neutrophils and lymphocytes during intestinal epithelium invasion by* Eimeria* [21].

Our qRT-PCR results revealed that the expression of MUC-2 was significantly reduced following infection. MUC-2 is the first line of innate host defence in preventing pathogen-induced epithelial injury [14]. PPE was able to alter this downregulation of MUC-2 due to infection, supporting the contention that PPE has a role in the regulation of goblet cell producing mucin [14].

*E. papillata* infection-induced status of oxidative damage to the jejuna tissue of mice [8, 10]. Nitric oxide pathway of inflammation intermediates was elevated as a consequence of the infection as revealed by increased activity of inducible nitric oxide synthase (iNOs) enzyme [22]. This finding also fits our data showing increased iNOS induced by infection. The inhibitory effect of pomegranate is mainly attributed to the impaired action of iNOS [23].

The jejuna of mice infected with* E. papillata* are characterized by inflammation, whose treatment with pomegranate appears to reduce. Indeed, pomegranate greatly influences the mRNAs of the inflammatory cytokines IL-1*β*, TNF-*α*, and INF-*γ* mRNA. Of these, IFN-*γ* is considered to be a major host defence mechanism against primary infections with* E. papillata* [24]. It has been suggested to be produced mainly by natural killer cells [24]. In this context, it is also noteworthy that pomegranate reduces the inflammatory response of the jejunum to infections with* E. papillata*.

Collectively, pomegranate exhibits a significant anticoccidial, antioxidant, and anti-inflammatory activities serving to protect against the tissue injuries induced by* Eimeria* and it is, therefore, highly recommended for use as a food additive in poultry farms.

## Figures and Tables

**Table 1:** *Eimeria papillata* developmental stages in jejuna of mice (per 10 villi) and oocyst output on day 5 p.i.

Group	Meronts	Male and female gamonts	Developing oocysts	Total number of parasitic stages	Oocyst output × 10^3^/g faeces
Infected (−PPE)	95 ± 11	64 ± 12	31 ± 5	190 ± 14	310 ± 47
Infected (+PPE)	58 ± 7^*^	31 ± 9^*^	21 ± 4^*^	110 ± 11^*^	140 ± 51^*^

Values are means ± SD.

^*^P ≤ 0.05, significant against infected (−PPE) group.

**Table 2:** Pomegranate ameliorates *E. papillata* induced changes in neutrophils and lymphocytes.

Group	Neutrophils (%)	Lymphocytes (%)
Noninfected (−PPE)	58 ± 4	35 ± 4
Noninfected (+PPE)	61 ± 3^*^	37 ± 3
Infected (−PPE)	80 ± 4^*^	17 ± 3^*^
Infected (+PPE)	69 ± 3^∗,∗∗^	26 ± 4^∗,∗∗^

Values are means ± SD.

^*^P ≤ 0.05, significant against noninfected (−PER) group; ^**^P ≤ 0.05, significant against infected (−PER) group.
